# Prescribing Alzheimer’s Disease treatments by provider type and geographic region: a comparison among physicians, nurse practitioners, and physician assistants

**DOI:** 10.1186/s12877-022-03176-3

**Published:** 2022-06-25

**Authors:** Jenny Y. Park, David L. Veenstra, Christopher J. Wallick, Zachary A. Marcum

**Affiliations:** 1 Department of Pharmacy, School of Pharmacy, University of Washington, Seattle, WA USA; 2 US Medical Affairs, Genentech, Inc, 1 DNA Way, South San Francisco, CA 94080 USA

**Keywords:** Alzheimer’s Disease, Dementia, Prescribing patterns, Provider type, Geographic disparities, Urban, Rural

## Abstract

**Background:**

The estimated increase in Alzheimer’s Disease (AD) caseload may present a logistical challenge to the US healthcare system. While nurse practitioners (NPs) and physician assistants (PAs) are increasingly delivering primary care to patients with chronic diseases, the nature of their prescribing of AD medications is largely unknown. The primary objective of this study was to compare the prescribing of AD medications across provider types (physician, NP, and PA) and geographic regions.

**Methods:**

We conducted a retrospective cohort study using IBM MarketScan® commercial and Medicare supplemental claims to examine unique AD prescriptions prescribed between January 1, 2016, and December 31, 2019. Parallel analysis of prescriptions for another geriatric condition, osteoporosis (OP), was also conducted for comparison.

**Results:**

A total of 103,067 AD prescriptions and 131,773 OP prescriptions were included in analyses. Physicians prescribed most AD prescriptions (95.65%), followed by NPs (3.37%) and PAs (0.98%). Small differences were identified among individual AD medications prescribed by physicians compared to NP/PAs. NPs/PAs prescribed a significantly higher proportion of AD prescriptions in rural as compared to urban areas (z = 0.023, 95%CI [0.018, 0.028]).

**Conclusion:**

Minimal variation exists in AD prescribing among physicians, NPs, and PAs, but NPs/PAs prescribe more AD prescriptions in rural areas. NPs/PAs, especially in rural areas, may play critical roles in alleviating projected workforce constraints. Further research assessing AD care, health outcomes, and costs by provider type and region is necessary to better guide healthcare workforce planning for AD care.

**Supplementary Information:**

The online version contains supplementary material available at 10.1186/s12877-022-03176-3.

## Background

Alzheimer’s Disease (AD) is a neurodegenerative disease currently ranked as the sixth leading cause of death in the United States (U.S.) [1]. Over six million Americans age 65 and older, roughly one in nine older adults, currently live with AD [1]. With the aging U.S. population, the number of patients with AD is expected to more than double to 13.5 million by 2050 [2].

AD may present a logistical challenge to the U.S. healthcare system [3]. The increasing number of patients with AD requires more diagnosing, prescribing, and monitoring for treatment and management. Although the workforce issues are multifaceted, many agree that the most pressing constraint is the inadequate supply of geriatrics and dementia specialists to treat patients [4]. Under current circumstances, a patient with dementia is projected to wait 18.6 months on average before receiving treatment [3]. Over 2.1 million patients with mild cognitive impairment (MCI) would develop AD during such a delay from 2020 to 2040 [3].

The recent approval of aducanumab along with the likely approval of disease modifying treatments (DMTs) in the future are expected to exacerbate projected workforce limitations. AD DMTs target amyloid or tau pathogenic pathways and are currently being studied in patients with MCI, early AD, and even preclinical AD. AD DMTs thus require earlier diagnoses. The primary safety concern with AD DMTs that are administered intravenously is amyloid-related imaging abnormalities (ARIA). AD DMTs therefore also require an increased need for brain imaging and biomarker testing, frequent monitoring for safety and efficacy, and specialists trained to prescribe them [3, 5, 6].

Nurse practitioners (NPs) and physician assistants (PAs) may play critical roles in alleviating wait times and providing access to care for patients with AD [7–9], especially in more rural areas where access to specialists is more limited [10]. In rural geographies from 2008 to 2016, the percentage of NPs (17.6% to 25.2%) and PAs (13.0% to 14.4%) serving as providers increased while that of physicians (69.4% to 60.5%) decreased [11]. Rural clinics in states with NP full scopes of practice had the highest percentages of practicing NPs practicing compared to states with restricted and reduced scopes of practice [11]*.*

Overall, NPs/PAs are increasingly delivering primary care to patients with chronic diseases [12, 13]. The extent to which NPs/PAs provide primary care for patients with AD is expected to vary by geographic region due to variation in state regulations regarding scope-of-practice [14, 15]. However, the extent and nature of current NP/PA involvement in treating AD remains largely unknown. Potential differences in NP and PA prescribing of AD treatments in relation to that of physicians (including specialists) are also unexamined.

## Objective

The primary objective of this study was to compare the prescribing of AD medications across provider types (physicians, NPs, and PAs) and geographic regions. The prescribing of AD medications by provider type and geographic region was also compared against that of osteoporosis (OP), another geriatric condition, for contextual comparison.

## Methods

### Study design and data source

A retrospective cohort study using medical and pharmacy claims was performed in the IBM MarketScan® Commercial Claims and Encounters and Medicare Supplemental databases [16]. The IBM MarketScan® Commercial Claims and Encounters and Medicare Supplemental databases are composed of de-identified patient-level health data regarding the annual medical utilization and expenditures for inpatient, outpatient, and prescription claims for over 90 million employees, their spouses, and their dependents who are covered under employer-sponsored private health insurance or Medicare supplemental insurance in the United States.

### Inclusion criteria

The focus of this analysis was on the prescription level. AD and OP prescription claims analyses included Food and Drug Administration (FDA) indicated AD (donepezil, galantamine, rivastigmine, memantine, and combination donepezil and memantine) and OP (abaloparatide, alendronate, alendronate and cholecalciferol, denosumab, ibandronate, raloxifene, risedronate, teriparatide, and zoledronic acid) prescriptions filled between 2016 to 2019. Inpatient prescriptions, duplicates, refills, and prescriptions for which the pharmacy fill date did not exactly match an outpatient service date were excluded for analysis. Prescriptions missing enrollee ID data, metropolitan statistical area (MSA) data, and provider type data were also excluded. Finally, prescriptions for which the listed provider type was an agency, facility, or a provider type lacking independent prescriptive authority were excluded.

Patient demographics (age and gender) and prescription characteristics (proportion of each drug prescribed and days supply) for included prescriptions were compared against all unique AD and OP prescriptions. Unique prescriptions were defined as prescriptions without missing data that were not refills or duplicates.

### Data collection: variables and measures

Due to a lack of provider type data in the pharmacy claims database within MarketScan, we obtained provider type data by tracing each pharmacy claim back to the enrollee’s nearest preceding outpatient service claim. We assumed that if an enrollee had visited an outpatient clinic on the same day that a unique prescription was filled for that enrollee at a pharmacy, then the provider seen at the outpatient clinic was most likely to have prescribed the unique prescription. Outpatient clinics, as defined by IBM MarketScan®, included hospital outpatient facilities, emergency rooms, and physician offices.

Similar methods were used to analyze AD and OP prescriptions. Specialist physicians were defined as neurologists for AD and endocrinologists or rheumatologists for OP.

We identified the total number and proportions of first observed original AD and OP prescriptions between 2016 and 2019 written by physicians, NPs, and PAs. The top five physician subtypes prescribing AD and OP prescriptions were also identified. Prescriptions were then grouped by provider type to describe the proportions of each medication prescribed by provider type. We compared the proportions of physicians, specialists, and NPs/PAs prescribing AD and OP medicines. We also compared the proportions of each medication prescribed by NPs/PAs against that of physicians and specialists (e.g., the proportion of donepezil prescribed by NPs/PAs compared to physicians).

### Regional analyses

The proportions of AD and OP medications prescribed by physician specialists, NPs, and PAs were assessed by metropolitan statistical area (MSA). To identify potential regional patterns of physician specialist prescribing, heat maps were generated to visually depict the proportion of medications in each MSA prescribed by physician specialists.

For rurality analyses, MSA-level data was divided into county-level data using the National Bureau of Economic Research (NBER) Census Core-Based Statistical Area (CBSA) to Federal Information Processing Series (FIPS) County Crosswalk [17]. Counties were then classified into one of six rurality categories using the National Center for Health Statistics (NCHS) Urban–Rural Classification Scheme for Counties [18]. MSAs not listed in the NBER crosswalk were excluded from rurality analyses.

For MSAs encompassing counties with different rurality categories, the most common rurality category within the MSA was set as the rurality category for the whole MSA. Because MSAs by definition require counties to be near an urbanized area with a population greater than 50,000, some US counties are not part of an MSA. Prescriptions written in rural areas that were not geographically classified into an MSA were separately placed into their own rurality category: rural. Including the pre-designated ‘rural’ category, each prescription was classified into one of seven total rurality categories. The rurality categories in the order of lowest to highest population density were rural, non-core, micropolitan, small metro, medium metro, large fringe metro, and large central metro. Rural and urban comparisons were made comparing rural counties to large fringe metro counties.

We identified and compared the proportion of prescriptions written by specialist physicians, NPs, and PAs in each rurality category. We also descriptively compared characteristics of the top 5% of counties with the highest proportion of prescriptions prescribed by NPs/PAs against the bottom 5% of counties with the lowest proportion of prescriptions prescribed by NPs/PAs.

### Statistical analyses

Due to the relatively smaller number of prescriptions written by NPs/PAs, prescriptions written by NPs/PAs were combined into one group (non-physician providers) for statistical analyses. Two-sample independent z-tests were performed to detect any statistically significant differences in the following outcomes: (1) the proportion of AD prescriptions prescribed by physicians, specialist physicians, and NPs/PAs (as compared to that of OP medications); (2) the proportion of individual AD and OP medications prescribed by physicians as compared to that prescribed by NPs/PAs; and (3) the proportions of specialist physicians and NPs/PAs prescribing amongst the rural and urban rurality categories. For all statistical analyses, we set alpha at 0.05 and obtained 95% confidence intervals (CI).

### Sensitivity analyses

A sensitivity analysis (SA) was conducted on the AD prescription cohort to further assess potential biases in our methodology of using same-day outpatient service claims to identify provider types prescribing AD treatments in pharmacy claims. In the SA, we increased the allowable time difference between the pharmacy fill-date and outpatient service date to 14-days; each prescription was traced back to the nearest preceding outpatient service date within 14 days of the fill date to identify provider type information. All other inclusion and exclusion criteria were kept the same as the original analyses.

### Software

SAS version 9.4 (SAS Institute Inc., Cary, NC) was used for constructing the analytic dataset and R version 4.0.2 (R Foundation for Statistical Computing, Vienna, Austria) for statistical analyses and generating heat maps [19, 20]. Microsoft PowerPoint version 16.49 and Microsoft Excel version 16.49 (Microsoft Corporation., Redmond, WA) were used to generate tables and figures.

## Results

### AD and OP prescription cohorts

**Fig. 1:**
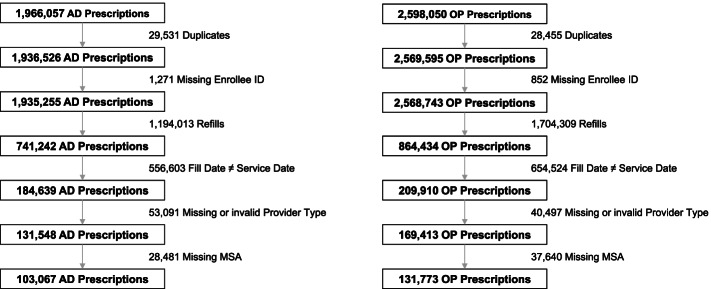
Cohort selection flowchart. Abbreviations: AD = Alzheimer’s Dementia, OP = Osteoporosis, ID = Identification, MSA = Metropolitan Statistical Area

Of 1,966,057 AD and 2,958,050 OP FDA-indicated prescriptions in MarketScan from 2016 to 2019, 5.24% (*N* = 103,067) AD and 4.45% (*N* = 131,773) of OP prescriptions were included for analysis (Fig. 1). The most common reasons for exclusion of AD and OP prescriptions first observed between 2016 and 2019 were due to the exclusion of refills (60.73% of AD prescriptions and 65.60% of OP prescriptions) and prescriptions for which the fill date did not exactly match an outpatient service date (28.31% of AD prescriptions and 25.19% of OP prescriptions).

Enrollees included in our analytic cohort for AD prescriptions were relatively younger as compared to enrollees of all unique AD prescriptions (Table 1). A higher proportion of donepezil and combination donepezil and memantine and a lower proportion of galantamine and memantine were observed in our analytic cohort as compared to all unique AD prescriptions. Patient demographics of our OP cohort were similar to that of all unique OP prescriptions. A higher proportion of alendronate and lower proportions of denosumab and teriparatide were observed in our analytic cohort as compared to all unique OP prescriptions.

**Table 1:** Characteristics of prescriptions included in analyses as compared to all prescriptions

Alzheimer’s Disease Cohort		
	**Included in Analysis**	**All Unique Prescriptions**
**Patient Demographics**		
Age: Mean (SD)	75.22 (13.91)	78.86 (12.43)
Gender (% Female)	57.64%	58.12%
**Prescriptions (n (%))**		
Donepezil	57,749 (56%)	375,576 (50%)
Galantamine	1,463 (1%)	14,210 (2%)
Rivastigmine	7,664 (8%)	61,521 (8%)
Memantine	34,555 (34%)	276,092 (37%)
Donepezil and Memantine	1,306 (1%)	1,935 (0.3%)
Total	103,067	744,180
Days Supply: Mean (SD)	44.71 (30.00)	42.37 (31.10)
**Osteoporosis Cohort**		
	**Included in Analysis**	**All Unique Prescriptions**
**Patient Demographics**		
Age: Mean (SD)	63.59 (10.32)	64.54 (10.70)
Gender (% Female)	92.66%	92.82%
**Prescriptions (n (%))**		
Abaloparatide	229 (0.2%)	2832 (0.4%)
Alendronate	80,539 (61%)	395,836 (56%)
Alendronate/Cholecalciferol	120 (0.1%)	964 (0.1%)
Denosumab	3,280 (2%)	36,970 (5%)
Ibandronate	18,585 (14%)	90,178 (13%)
Raloxifene	16,704 (13%)	102,461 (14.5%)
Risedronate	10,766 (8%)	57,811 (8%)
Teriparatide	1,362 (1%)	17,624 (2%)
Zoledronic Acid	185 (0.1%)	1,734 (0.25%)
Romosozumab-aqqg	3	35
Total	131,773	706,445
Days Supply: Mean (SD)	60.65 (33.67)	62.72 (37.21)

*Abbreviations:* *SD* Standard Deviation

### Provider types prescribing AD and OP medications

Table 2 depicts the proportion of AD and OP prescriptions written by NPs, PAs, physicians, and physician specialists. Physicians and NPs/PAs prescribed 95.65% and 4.34% of AD prescriptions, respectively. NPs/PAs prescribed a statistically significantly higher proportion of AD prescriptions as compared to OP prescriptions. The five physician specialties prescribing the largest proportions of AD prescriptions were family practice, neurology, internal medicine, multispecialty, and psychiatry. Physician specialists prescribed 23.21% of AD prescriptions, which was statistically significantly higher than the proportion of OP prescriptions prescribed by physician specialists (10.29%, (z = 0.188, 95% CI [0.185, 0.191]).

**Table 2:** The proportion of AD and OP prescriptions written by NPs, PAs, Physicians and Physician subtypes

Alzheimer’s Disease Prescriptions		
	**Count**	**Percentage of All Prescriptions**
**Physicians**	**98,588**	**95.65%**
Family Practice	29,655	28.77%
Neurology	23,924	23.21%
Internal Medicine	16,254	15.77%
Multispecialty	8,867	8.60%
Psychiatry	5,459	5.30%
Geriatric Medicine^a^	813	0.79%
Other	13,616	13.21%
**Non-Physician Professionals**	**4,479**	**4.35%**
Nurse Practitioner	3,477	3.37%
Physician Assistant	1,002	0.98%
**Total**	**103,067**	**100%**
**Osteoporosis Prescriptions**		
	**Count**	**Percentage of All Prescriptions**
**Physicians**	**126,836**	**96.25%**
Family Practice	39,137	29.70%
Internal Medicine	30,005	22.77%
Obstetrics & Gynecology	10,179	7.72%
Rheumatology	7,804	5.92%
Multispecialty	6,183	4.69%
Endocrinology & Metabolism^a^	5,763	4.37%
Geriatric Medicine^a^	194	0.15%
Other	27,571	20.92%
**Non-Physician Professionals**	**4,937**	**3.75%**
Nurse Practitioner	3,465	2.63%
Physician Assistant	1,472	1.12%
**Total**	**131,773**	**100%**

*Abbreviations:* *AD* Alzheimer’s Disease, *NP* Nurse Practitioner, *PA* Physician Assistant

^a^ Not top five but relevant and important

**Table 3:** Comparing county characteristics between the top 5% and bottom 5% of counties with the highest and lowest proportion of prescriptions prescribed by NPs and PAs

	Top 5%	Bottom 5%
Percent of all prescriptions written by NPs and PAs	17.30%	0.9%
County population	136,103	680,809
Median household income	$62,642	$67,509

*Abbreviations*: *NPs* Nurse Practitioners, *PAs* Physician Assistants

### AD and OP medications prescribed by provider type

**Fig. 2:**
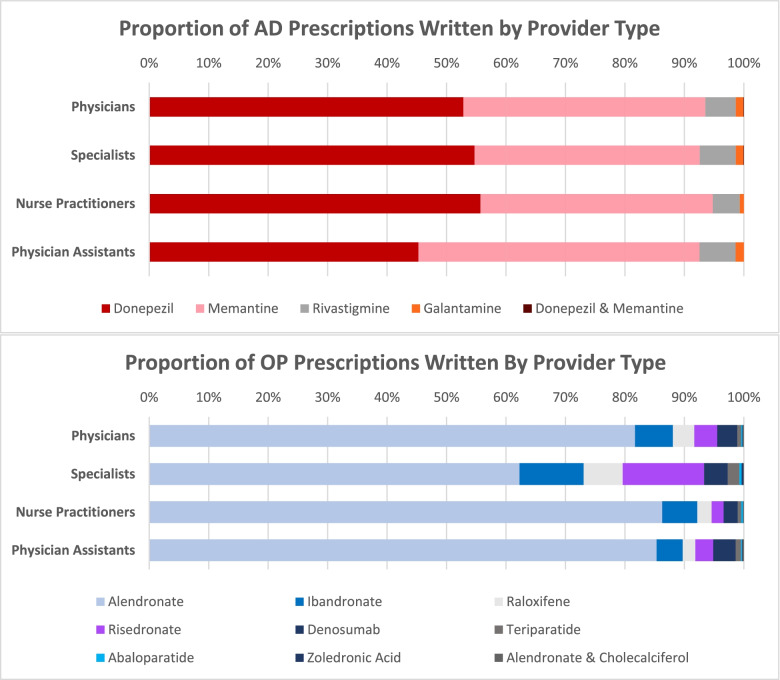
Proportions of AD and OP medicines prescribed by NPs, PAs, and Physicians. Abbreviations: AD = Alzheimer’s Dementia, OP = Osteoporosis, NP = Nurse Practitioner, PA = Physician Assistant

The proportions of individual AD and OP medications prescribed by NPs, PAs, and physicians are presented in Fig. 2. Overall, donepezil was the most frequently prescribed AD medication followed by memantine, rivastigmine, galantamine, and combination donepezil and memantine. Physicians prescribed significantly higher proportions of donepezil (z = 0.056, 95% CI [0.041, 0.071]) and significantly lower proportions of memantine (z = -0.045, 95% CI [-0.059, -0.030]) and combination donepezil and memantine (z = -0.011, 95% CI [-0.014, -0.008]) as compared to NPs/PAs. Specialists did not significantly differ from NPs/PAs in the AD medicines they prescribed; however, OP specialists prescribed significantly lower proportions of alendronate (z = -0.237, 95% CI[-0.254, -0.220]) and significantly higher proportions of ibandronate (z = 0.053, 95% CI[0.042, 0.064]), raloxifene (z = 0.058, 95% CI[0.065, 0.051]), risedronate (z = 0.113, 95% CI[0.103, 0.123]), and teriparatide (z = 0.013, 95% CI[0.008, 0.017]).

Alendronate was the most frequently prescribed OP medication followed by ibandronate, raloxifene, risedronate, denosumab, teriparatide, abaloparatide, zoledronic acid, and combination alendronate and cholecalciferol. Compared to NPs/PAs, physicians prescribed significantly higher proportions of alendronate (z = 0.032, 95% CI [0.018, 0.046]) and significantly lower proportions of raloxifene (z = -0.013, 95% CI [-0.022, -0.004]) and risedronate (z = -0.018, 95% CI [-0.025, -0.011]).

### Regional analyses: AD and OP prescriptions

**Fig. 3:**
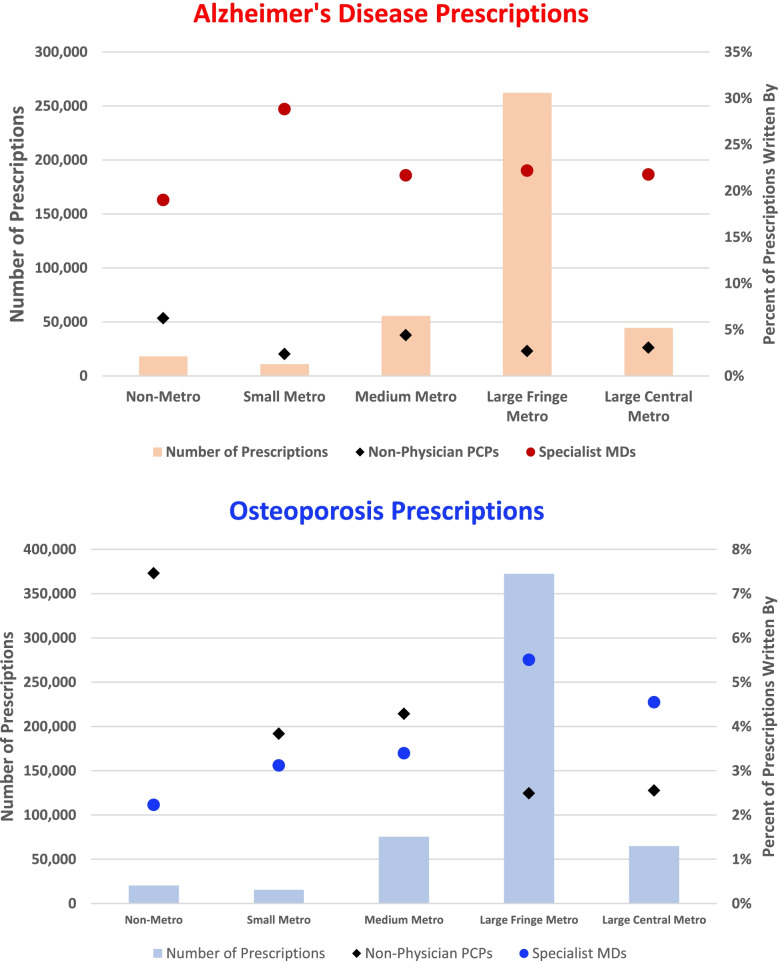
The distribution of prescriptions and proportion of prescriptions written by non-physician PCPs (NPs/PAs) or specialist physicians by rurality. Abbreviations: PCP = Primary Care Provider, NP = Nurse Practitioner, PA = Physician Assistants, MD = Medical Doctor. The Non-Metro category incluedes rural and micropolitan counties

A total of 90,657 (88%) AD prescriptions and 115,331 (87.5%) OP prescriptions were included in regional analyses after excluding prescriptions with MSAs not listed in the NBER crosswalk. AD and OP prescriptions were similarly distributed across the rurality categories (Fig. 3). Most of the AD (32.41%) and OP (37.45%) prescriptions were filled in large fringe metros. Very few prescriptions were filled in micropolitan areas.

NPs/PAs prescribed a significantly higher proportion (z = 0.023, 95% CI [0.018, 0.028]) of AD prescriptions in rural MSAs (6.32%) as compared to urban MSAs (2.68%). The proportion of AD prescriptions prescribed by physician specialists did not significantly differ between rural (18.8%) and urban (22.20%) areas.

**Fig. 4:**
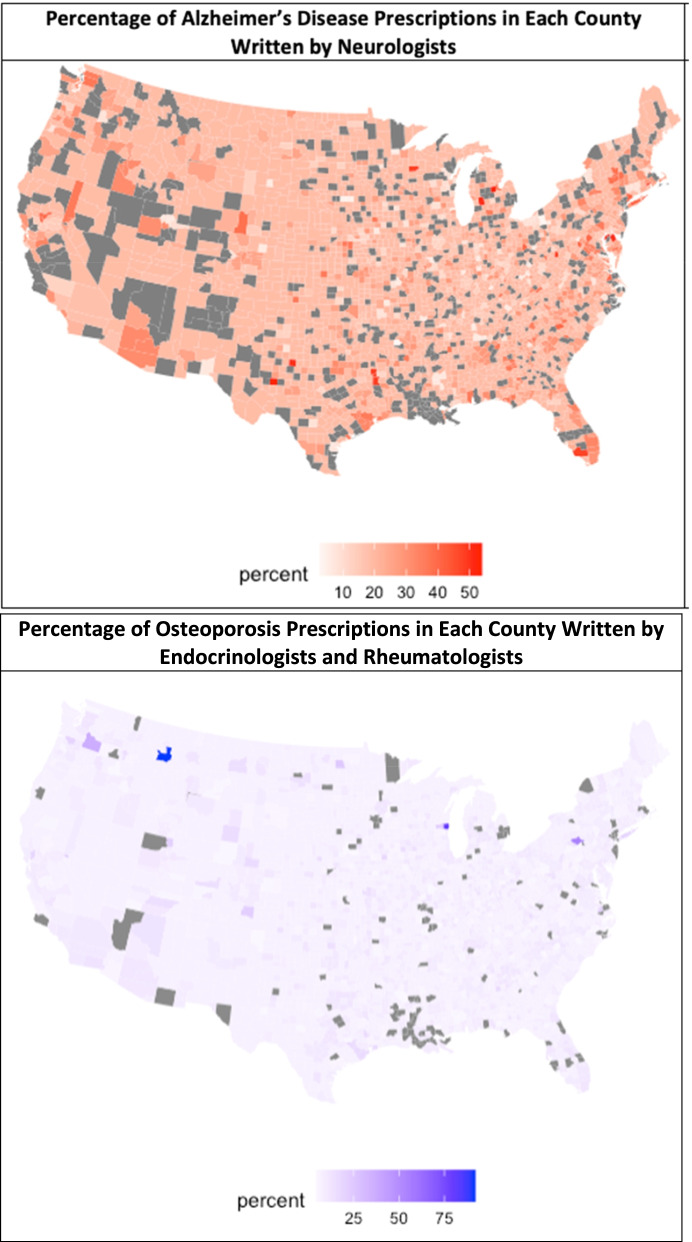
Heat maps depicting the proportion of prescriptions prescribed by specialist physicians in each county

In the top 5% of counties with the highest proportion of AD prescriptions prescribed by NPs/PAs, NPs and PAs prescribed 17.30% of all AD prescriptions (Fig. 4). In the bottom 5% of counties with the lowest proportion of AD prescriptions prescribed by NPs/PAs, NPs and PAs prescribed 0.9% of all AD prescriptions. The counties in the top 5% had smaller county populations and slightly lower median household incomes as compared to the bottom 5% (Table 3).

NPs/PAs prescribed a significantly higher proportion (z = 0.067, 95% CI [0.064, 0.069]) of OP prescriptions in rural MSAs (7.74%) as compared to urban MSAs (2.49%). Physician specialists prescribed a significantly lower proportion (z = 0.032, 95% CI [0.027, 0.036]) of OP prescriptions in rural MSAs (1.93%) as compared to urban MSAs (5.51%).

Heat maps were generated depicting the proportion of AD and OP prescriptions written by specialists in each county (Fig. 4). Visual inspection of the maps revealed no apparent geographic variation in the proportion of specialists prescribing AD medications across counties; similarly, no apparent geographic variation was evident regarding the proportion of specialists prescribing OP medications.

### Sensitivity analyses

Increasing the provider type identification time window from same-day to 14-days resulted in the inclusion of 193,064 more AD prescriptions in the analytical cohort as compared to our original approach; in the SA, the cohort increased by 187% to a total of 296,131 prescriptions included in analyses.

Table 4 depicts the proportion of AD prescriptions written by NPs, PAs, physicians, and physician specialists in the SA cohort. Physicians and NPs/PAs prescribed 95.27% and 4.73% of AD prescriptions, respectively. The top five physician subtypes prescribing AD treatments in the SA cohort were family practice, internal medicine, multispecialty, radiology, and podiatry. Physician specialists wrote 4.27% of AD prescriptions and were not included in the top five physician subtypes prescribing AD treatments.

**Table 4:** Sensitivity Analysis

**Alzheimer’s Disease Prescriptions (14-day time window)**		
	**Count**	**Percentage of All Prescriptions**
**Physicians**	**282,127**	**95.27%**
Family Practice	82,482	27.85%
Internal Medicine	40,600	13.71%
Multispecialty	29,516	9.97%
Radiology	16,113	5.44%
Podiatry	15,396	5.20%
Neurology^a^	12,643	4.27%
Other	85,377	28.83%
**Non-Physician Professionals**	**14,004**	**4.73%**
Nurse Practitioner	10,367	3.50%
Physician Assistant	3,637	1.23%
**Total**	**296,131**	**100%**
**Alzheimer’s Disease Prescriptions (Same day)**		
**Physicians**	**98,588**	**95.65%**
Family Practice	29,655	28.77%
Neurology	23,924	23.21%
Internal Medicine	16,254	15.77%
Multispecialty	8,867	8.60%
Psychiatry	5,459	5.30%
Other	14,429	14.0%
**Non-Physician Professionals**	**4,479**	**4.35%**
Nurse Practitioner	3,477	3.37%
Physician Assistant	1,002	0.98%
**Total**	**103,067**	**100%**

^a^Not one of the top 5 physician subtypes identified but included separately due to relevance (AD specialists)

## Discussion

This study descriptively compared prescribing of medications for AD among three provider types – physicians, NPs, and PAs – and examined regional variation of prescribing AD medications. Statistically significant differences in the proportions of some AD medications (i.e., donepezil, memantine, and combination donepezil and memantine) prescribed by NPs/PAs and physicians were identified; however, these differences were numerically small and unlikely to be clinically meaningful. When using the geriatric condition of osteoporosis for contextual comparison, NPs/PAs and physician specialists prescribed significantly higher proportions of AD prescriptions as compared to OP prescriptions, suggesting greater involvement in the care of AD. NPs/PAs prescribed a significantly higher proportion of AD prescriptions in rural areas as compared to urban areas.

The statistically significant differences in the proportion of AD and OP prescriptions written by NPs/PAs in rural compared to urban geographies are likely attributable to the increasing volume of NPs/PAs serving as primary care providers in rural areas [11]. Counties with the highest and lowest proportions of prescriptions prescribed by NPs and PAs differed in county population and medium household incomes. Further research may elucidate other differences between counties with varying involvement of NPs and PAs managing patients with AD.

### Limitations

These results should be interpreted in the context of important limitations. First, selection bias may be a concern as included prescriptions represented roughly 5% of all AD and OP prescriptions identified from MarketScan during this time period. As such, our results can be generalized to unique prescriptions for AD, not refills.

We excluded prescriptions for which the fill date was not traceable to a same-day outpatient clinic service date in efforts to maximize the internal validity of our provider type analyses while minimizing risk of incorrect provider type identification. By requiring the same-day fill date and outpatient service date, our analyses may include more prescriptions from institutions with an on-site pharmacy or automated order entry processes. However, the SA elucidates the heightened risk of incorrect provider type matching that occurs when loosening the time window to match prescriptions to outpatient service dates. Podiatrists and radiologists are unlikely to be top prescribers of AD treatments. The SA thus further substantiates the validity of retaining the same-day methodology to identify provider types.

There were some minor differences in age of enrollees and proportions of certain medications when comparing included versus excluded prescriptions. However, these differences were minor and unlikely to significantly affect the internal validity of our results. Additional research in a dataset with provider type information included in prescription claims data may alleviate selection bias concerns while preserving data integrity. Overall, our results represent the analysis of original prescriptions to characterize existing geographic differences regarding access to certain provider types.

The lack of provider type data in the pharmacy claims database within MarketScan may have resulted in the incorrect matching of provider data for some prescriptions. Incorrectly matching provider data to prescriptions would likely reduce the internal validity of our results. To minimize the risk of incorrectly matching providers to prescriptions, we used the most conservative method of only including prescriptions for which the pharmacy fill date was the same day as a documented outpatient service claims date for the same enrollee.

A subset of the prescriptions included in our analytic cohort may have been prescribed by a NP or PA but billed under a physician national provider identifier (NPI) under certain conditions for reimbursement purposes. Issues regarding incident-to-billing would lead to an underrepresentation of NPs/PAs in our results as compared to their actual volume of prescribing for patients with AD and OP. While the exact frequency of incident-to-billing remains unclear, our results must be interpreted with awareness that NPs/PAs may be underrepresented in our analysis.

Regional analyses may be limited by the granularity of geographic data in MarketScan. Some counties may have been miscategorized into incorrect rurality categories due to potential errors while converting MSA-level data into county-level data. Using the well-established NBER CBSA crosswalk for the conversion of MSA-level data into county-level data likely led to the correct categorization of most counties and hence maximized the internal validity of our regional analyses.

Finally, our analytic cohort included data from those with employer-sponsored supplemental Medicare coverage, which is representative of 29% of all Medicare beneficiaries [21]. Our analytic cohort thus likely includes prescriptions for patients with AD skewed to represent slightly younger patients compared to the general population of all patients with AD. The younger population in our sample are more likely to have less advanced disease and fewer comorbidities compared to the average population; they may receive a higher proportion of care from NPs/PAs. While further analyses including the Medicare fee-for-service (FFS) beneficiaries will be beneficial, our results may still provide a valuable summary of current prescribing of AD treatments considering the large number of prescriptions captured in the analyses.

### Implications

While a statistically significantly higher proportion of prescriptions were written by NPs/PAs in the AD cohort (4.35%) as compared to the OP cohort (3.75%), the proportion of AD prescriptions prescribed by NPs/PAs is very small compared to that of other diseases. For example, approximately 21.8% of patients with diabetes are managed by NPs/PAs [15], and 23% of antibiotics are prescribed by NPs/PAs [22]. Since 2000, the number of geriatricians per 10,000 adults 65 years and older has decreased [23]; the U.S. Department of Health and Human Services suggests that increasing the number of NPs and PAs to provide primary care can alleviate the projected shortage of physicians for managing AD [8, 24].

Recent scope-of-practice regulations allowing NPs and PAs to prescribe without physician oversight has demonstrated positive impacts on costs and patient outcomes. Practitioner labor costs per visit along with total labor costs per visit are significantly lower among practices with greater NP and PA involvement as primary care providers [25]. In one study, patients taking anti-diabetic medications had significantly higher medication adherence rates and probabilities of good adherence in states that expanded NP scope of practice [26]. In a systematic literature review, three studies reported significantly greater primary care access in states where NPs have full practice authority [15].

Granting NPs and PAs more consistent authority to enable full practice when managing patients with chronic conditions may alleviate projected workforce constraints and delays in access to care. Further training may be required to enhance the workforce, including NPs and PAs, to provide care for patients with AD. Interprofessional dementia education has been shown to successfully enhance NP’s basic competency in the detection and management of AD [27]. Specialized training, additional certifications, and continuing education programs can also improve the quality of dementia care NPs and PAs provide [24]. Policymakers, academic institutions, and health systems may play critical roles in providing access to and raising awareness of such training opportunities.

With the aging population of the U.S., there will be an increased need for diagnosing, prescribing, and monitoring for patients with AD. Roughly 16% of the U.S. older adult population is expected to have AD in 2050 [2], and proactive solutions are necessary to ensure timely and quality care for older adults.

Moreover, the recent approval of aducanumab, along with the expected approval of AD DMTs in the future, will likely change the infrastructure of AD care in the U.S. The need for early diagnosing and treatment will likely increase with the approval of treatments intended for patients with mild AD [28]. The increased need for monitoring coupled with more frequent brain imaging needs are also expected with amyloid-beta and tau targeting treatments. Such changes may exacerbate already existing challenges and barriers accessing AD specialists, especially in rural geographies. While care patterns for screening, diagnosis, treatment, and follow up for patients with AD are changing, a clear understanding of current prescribing practice can provide a helpful “benchmark” for future planning of AD care.

## Conclusion

We conducted a retrospective cohort study using insurance claims data and descriptively assessed the prescribing of AD treatments by provider type and geographic region. NPs/PAs prescribe roughly 4% of AD prescriptions. Minimal variation exists in AD prescribing among physicians, NPs, and PAs. However, NPs/PAs prescribe significantly more in rural areas as compared to urban areas.

Greater involvement of NPs and PAs in the care of patients with AD may help alleviate projected workforce constraints and delays in access to care for patients with AD. Further research identifying current AD care patterns and comparing AD health outcomes and costs by provider type and geographic region may be necessary to more effectively increase the capacity of our current and future workforce to provide timely care for patients with AD.

## Supplementary Information

**Additional file 1:** **Appendix 1.** Visual Representation of Table 2.  **Appendix 2.** Data for Figure 2. **Appendix 3.** Data for Figure 3. **Appendix 4.** Regional Top and Bottom Analysis. **Appendix 5.** Results of the 2-sample independent z-tests. 

## Data Availability

The data that support the findings of this study are available from IBM MarketScan® but restrictions apply to the availability of these data, which were used under license for the current study, and so are not publicly available. Data are however available from the authors upon reasonable request and with permission of IBM MarketScan®. Contact Jenny Park, the corresponding author, to request data from this study.

## Abbreviations

AD: Alzheimer’s Disease
NP: Nurse Practitioner
PA: Physician Assistant
OP: Osteoporosis
US: United States
MCI: Mild Cognitive Impairment
DMT: Disease Modifying Treatment
ARIA: Amlyoid Related Imaging Abnormalities
FDA: Food and Drug Administration
MSA: Metropolitan Statistical Area
NBER: National Bureau of Economic Research
CBSA: Census Core-Based Statistical Area
FIPS: Federal Information Processing Series
NCHS: National Center for Health Statistics
CI: Confidence Interval
ID: Identification
SD: Standard Deviation
PCP: Primary Care Provider
MD: Medical Doctor
NPI: National Provider Identifier
FFS: Fee-for-service
