# Supplementary material for: Prescribing Alzheimer’s Disease treatments by provider type and geographic region: a comparison among physicians, nurse practitioners, and physician assistants
Source: BMC Geriatr. 2022 Jun 25;22:522. doi: 10.1186/s12877-022-03176-3 (PMC9233396; doi:10.1186/s12877-022-03176-3)
Supplement: Supplementary file 1 — Additional file 1: Appendix 1. Visual Representation of Table 2. Appendix 2. Data for Figure 2. Appendix 3. Data for Figure 3. Appendix 4. Regional Top and Bottom Analysis. Appendix 5. Results of the 2-sample independent z-tests. [file 12877_2022_3176_MOESM1_ESM.docx]

**Appendix**

| Appendix 1: Visual Representation of Table 2 |
| --- |
| 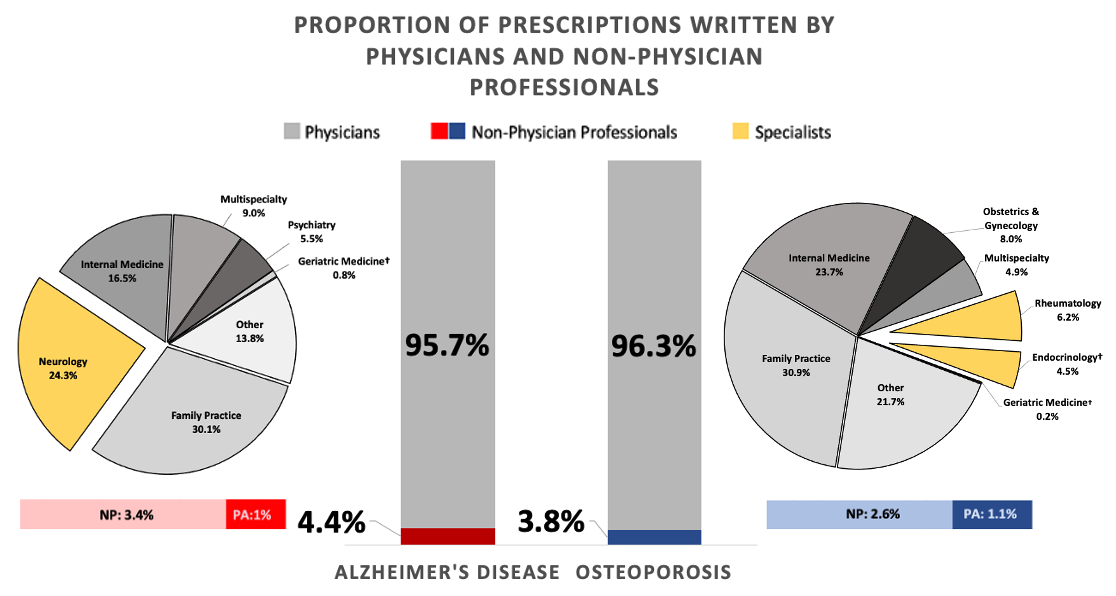 |
| Abbreviations: NPs = Nurse Practitioners, PAs = Physician Assistants |

| Appendix 2: Data for Figure 2 |
| --- |
| \| Osteoporosis \| \| \| \| \| \| \| \| \| \| \| --- \| --- \| --- \| --- \| --- \| --- \| --- \| --- \| --- \| --- \| \|  \| Alendronate \| Ibandronate \| Raloxifene \| Risedronate \| Denosumab \| Teriparatide \| Abaloparatide \| Zoledronic Acid \| Alendronate & Cholecalciferol \| \| PA \| 63.38% \| 13.86% \| 9.99% \| 8.02% \| 2.79% \| 1.70% \| 0.14% \| 0 \| 0.14% \| \| NP \| 64.53% \| 14.66% \| 12.01% \| 5.77% \| 1.76% \| 0.95% \| 0.23% \| 0.03% \| 0.06% \| \| MD \| 61% \| 14.09% \| 12.73% \| 8.24% \| 2.51% \| 1.03% \| 0.17% \| 0.15% \| 0.09% \|  \| Alzheimer’s Disease \| \| \| \| \| \| \| --- \| --- \| --- \| --- \| --- \| --- \| \|  \| Donepezil \| Memantine \| Rivastigmine \| Galantamine \| Donepezil & Memantine \| \| PA \| 50.90% \| 40.22% \| 7.29% \| 1.60% \| 0.00% \| \| NP \| 52.14% \| 39.09% \| 8.02% \| 1.38% \| 1.41% \| \| MD \| 57.47% \| 33.34% \| 7.74% \| 1.42% \| 0.03% \| |
| Abbreviations: NP = Nurse Practitioner, PA = Physician Assistants, MD = Medical Doctor |

| Appendix 3: Data for Figure 3 |
| --- |
| \| **Rurality**  **(NCHS)** \| **Alzheimer’s Disease** \| \| \| \| **Osteoporosis** \| \| \| \| \| --- \| --- \| --- \| --- \| --- \| --- \| --- \| --- \| --- \| \| **Total** \| **Specialists** \| **NP** \| **PA** \| **Total** \| **Specialists** \| **NP** \| **PA** \| \| **MSA = 0** \| 19638 \| 4292 \| 858 \| 289 \| 18554 \| 1290 \| 1026 \| 411 \| \| **1** \| 8906 \| 2038 \| 221 \| 96 \| 25093 \| 959 \| 205 \| 120 \| \| **2** \| 33402 \| 7986 \| 910 \| 236 \| 49351 \| 6217 \| 907 \| 395 \| \| **3** \| 20064 \| 4318 \| 776 \| 237 \| 25093 \| 2278 \| 764 \| 311 \| \| **4** \| 8643 \| 1804 \| 359 \| 67 \| 10363 \| 830 \| 370 \| 120 \| \| **5** \| 4 \| 1 \| 1 \| 0 \| 11 \| 4 \| 1 \| 0 \| \| **6** \| 0 \| 0 \| 0 \| 0 \| 0 \| 0 \| 0 \| 0 \| \| **Excluded*** \| 12410 \| \| \| \| 16442 \| \| \| \| \| *MSA not listed in the Census Bureau Crosswalk  National Center for Health Statistics (NCHS) Rurality Classification: (1: Large central metro, 2: Large fringe metro, 3: Medium Metro, 4: Small Metro, 5: Micropolitan, 6: Non-Core) \| \| \| \| \| \| \| \| \| \| Abbreviations: NCHS = National Center for Health Statistics, NP = Nurse Practitioner, PA = Physician Assistant \| \| \| \| \| \| \| \| \| |

| Appendix 4: Regional Top and Bottom Analysis |
| --- |
| \|  \| AD Top 5% \| \| AD Bottom 5% \| \| \| --- \| --- \| --- \| --- \| --- \| \|  \| Mean \| SD \| Mean \| SD \| \| Percent NP/PA \| 17.30% \| 2.41 \| 0.9% \| 0.04 \| \| County Population \| 135,103 \| 173,109 \| 680,809 \| 1,923,149 \| \| Median Household Income \| $62,642 \| 8,909 \| $67,509 \| 15716 \| \| Unemployment \| 3.57% \| 0.76 \| 3.37% \| 0.51 \| \| % High School \| 9.4% \| 4.1 \| 12.86% \| 4.33 \| \| % College \| 30.20% \| 11.6 \| 27.91% \| 12.67 \| |
| Abbreviations: AD = Alzheimer’s Disease, SD = Standard Deviation, NP = Nurse Practitioner, PA = Physician Assistant |

| Appendix 5: Results of the 2-sample independent z-tests |
| --- |
| \| **Test** \| **Coefficient** \| **Result (p-value and 95% CI)** \| \| --- \| --- \| --- \| \| Provider type proportions: AD compared to OP \| \| \| \| Specialists* \| 0.188 \| p-value < 0.001 \| (0.185, 0.191) \| \| Nurse Practitioners* \| 0.007 \| p-value < 0.001 \| (0.006, 0.008) \| \| Physician Assistants* \| -0.004 \| p-value < 0.001 \| (-0.002, -0.0006) \| \| NPs/PAs* \| -0.0013 \| p-value < 0.001 \| (0.004, 0.008) \| \| Medicines prescribed: physicians compared to NPs/PAs \| \| \| \| Donepezil* \| 0.056 \| p-value < 0.001 \| (0.041, 0.071) \| \| Memantine* \| -0.0445 \| p-value < 0.001 \| (-0.059, -0.030) \| \| Rivastigmine \| -0.001 \| p-value = 0.815 \| (-0.009, 0.007) \| \| Galantamine \| 0 \| p-value = 1 \| (-0.004, 0.004) \| \| Donepezil & Memantine* \| -0.011 \| p-value < 0.001 \| (-0.014, -0.008) \| \| Medicines prescribed: physicians compared to NPs/PAs \| \| \| \| Alendronate* \| 0.032 \| p-value < 0.001 \| (0.018, 0.046) \| \| Ibandronate \| 0.003 \| p-value = 0.527 \| (-0.007, 0.013) \| \| Raloxifene* \| -0.013 \| p-value = 0.007 \| (-0.022, -0.004) \| \| Risedronate* \| -0.018 \| p-value < 0.001 \| (-0.025, -0.011) \| \| Denosumab \| -0.0046 \| p-value =0.058 \| (-0.009, -0.0002) \| \| Teriparatide \| 0.011 \| p-value = 0.3534\| (0.012, 0.010) \| \| Abaloparatide \| 0.0005 \| p-value = 0.738 \| (-0.001, 0.002) \| \| Zoledronic Acid \| -0.0005 \| p-value = 0.035 \| (-0.002, -0.001) \| \| Alendronate & Cholecalciferol \| 0.001 \| p-value = 1 \| (0.001, 0.001) \| \| Rural vs. Large Fringe Metro \| \| \| \| AD Non-Physician PCPs* \| 0.023 \| p-value < 0.001 \| (0.018,0.028) \| \| AD Nurse Practitioners* \| 0.0175 \| p-value < 0.001 \| (0.014, 0.023) \| \| AD Physician Assistants* \| 0.004 \| p-value = 0.009 \| (0.001, 0.007) \| \| AD Specialists \| -0.0104 \| p-value = 0.055 \| (-0.021, 0.0002) \| \| OP Non-Physician PCPs* \| 0.0665 \| p-value < 0.001 \| (0.064, 0.069) \| \| OP Nurse Practitioners* \| 0.0472 \| p-value < 0.001 \| (0.0434, 0.051) \| \| OP Physician Assistants* \| 0.0035 \| p-value = 0.009 \| (0.002,0.005) \| \| OP Specialists* \| 0.0315 \| p-value = 0.001 \| (0.027, 0.036) \| \| *: Significant at alpha=0.05 \| \| \| |
| Abbreviations: CI = Confidence Interval, AD = Alzheimer’s Disease, OP = Osteoporosis, NP = Nurse Practitioner, PA = Physician Assistant |
